# Can metformin relieve tibiofemoral cartilage volume loss and knee symptoms in overweight knee osteoarthritis patients? Study protocol for a randomized, double-blind, and placebo-controlled trial

**DOI:** 10.1186/s12891-022-05434-2

**Published:** 2022-05-21

**Authors:** Guangfeng Ruan, Shiwen Yuan, Aiju Lou, Yingqian Mo, Yuan Qu, Dongmei Guo, Shangqi Guan, Yan Zhang, Xiaoyong Lan, Jun Luo, Yifang Mei, Hongwei Zhang, Weirong Wu, Lie Dai, Qinghong Yu, Xiaoyan Cai, Changhai Ding

**Affiliations:** 1 Clinical Research Centre, Guangzhou First People’s Hospital, School of Medicine, South China University of Technology, Guangzhou, China; 2 Clinical Research Centre, Zhujiang Hospital, Southern Medical University, Guangzhou, Guangdong China; 3 Department of Rheumatology, Guangzhou First People’s Hospital, School of Medicine, South China University of Technology, Guangzhou, Guangdong China; 4 Department of Rheumatology and Immunology, Liwan Central Hospital of Guangzhou, Guangzhou, Guangdong China; 5 Department of Rheumatology, Sun Yat-Sen Memorial Hospital, Sun Yat-Sen University, Guangzhou, Guangdong China; 6 Rheumatology and Clinical Immunology Department, Zhujiang Hospital, Southern Medical University, Guangzhou, Guangdong China; 7 Department of Rheumatology, Foshan First People’s Hospital, Foshan, Guangdong China; 8 Department of Rheumatology, Third People’s Hospital of Shenzhen, The Second Affiliated Hospital of Southern University of Science and Technology, Shenzhen, Guangdong China; 9 Department of Rehabilitation Medicine, the Second Affiliated Hospital of Nanchang University, Nanchang, Jiangxi China

**Keywords:** Knee osteoarthritis, Metformin, Randomized controlled trial

## Abstract

**Background:**

Osteoarthritis (OA) is the most common joint disease, and is most frequently seen in the knees. However, there is no effective therapy to relieve the progression of knee OA. Metformin is a safe, well-tolerated oral medication that is extensively used as first-line therapy for type 2 diabetes. Previous observational studies and basic researches suggested that metformin may have protective effects on knee OA, which needs to be verified by clinical trials. This study, therefore, aims to examine the effects of metformin versus placebo on knee cartilage volume loss and knee symptoms in overweight knee OA patients by a randomized controlled trial over 24 months.

**Methods:**

This protocol describes a multicenter, randomized, double-blind, and placebo-controlled clinical trial aiming to recruit 262 overweight knee OA patients. Participants will be randomly allocated to the two arms of the study, receiving metformin hydrochloride sustained-release tablets or identical inert placebo for 24 months (start from 0.5 g/day for the first 2 weeks, and increase to 1 g/day for the second 2 weeks, and further increase to 2 g/day for the remaining period if tolerated). Primary outcomes will be changes in tibiofemoral cartilage volume and Western Ontario and McMaster Universities Osteoarthritis Index (WOMAC) score over 24 months. Secondary outcomes will be changes in visual analogue scale (VAS) knee pain, tibiofemoral cartilage defects, effusion-synovitis volume, and tibiofemoral bone marrow lesions maximum size over 24 months. The primary analyses will be intention-to-treat analyses of primary and secondary outcomes. Per-protocol analyses will be performed as the secondary analyses.

**Discussion:**

If metformin is proved to slow knee cartilage volume loss and to relieve knee symptoms among overweight knee OA patients, it will have the potential to become a disease modifying drug for knee OA. Metformin is a convenient intervention with low cost, and its potential effects on slowing down the structural progression and relieving the symptoms of knee OA would effectively reduce the disease burden worldwide.

**Trial registration:**

ClinicalTrials. gov NCT05034029. Registered on 30 Sept 2021.

## Background

Osteoarthritis (OA) is one of the most common joint diseases worldwide, and knee is the most commonly affected joint [1]. It leads to joint pain and physical disability, decreases mobility and quality of life, and is the major cause of the increasing demand for joint replacement [2–4]. Currently, more than 300 million people suffering from OA worldwide [5]. In the population over 50 years of age, the prevalence of OA is up to 10-20% [6]. OA has a huge economic burden, and its social cost could account for 0.25-0.5% of a country’s gross domestic product (GDP) [7]. Moreover, due to the increased proportion of the elderly and obese people in the population, the incidence of OA is expected to increase further [3]. However, there has been no disease-modifying OA drug approved by the regulatory bodies at present [2]. Thus, there is an urgent need to find drugs that can effectively modify the progression of OA.

Metformin is a safe, well-tolerated oral medication that is widely used as the first-line therapy for type 2 diabetes [8]. In recent years, metformin has attracted extensive attention for its potentially multiple effects such as weight reduction, anti-inflammatory, anti-oxidant, and anti-aging [9–12]. Given the biological effects of metformin and the important roles of overweight, inflammation, oxidative stress, and aging playing in knee OA, metformin might be a potential disease modifying agent for knee OA, especially for those with the obese phenotype [13].

Indeed, accumulating evidences suggest that metformin may have OA protective effects. A study reported that metformin administered intragastrically in destabilization of the medial meniscus (DMM) mouse model of knee OA could maintain the balance of chondrocyte synthesis and catabolism, delay cartilage aging and the progression of knee OA by regulating the AMP-activated protein kinase (AMPK) /mammalian target of rapamycin (mTOR) signaling pathway [14]. Similar to this study, another study demonstrated that intraarticular injections of metformin in DMM-induced OA mice mitigated cartilage degradation by activating AMPK/the specifically silent information regulator 1 (SIRT1)-mediated autophagy [15]. Besides, the cartilage protective effect of metformin was also confirmed in both collagenase-induced and papain-induced OA mouse models [16, 17]. Three other in vivo studies demonstrated that metformin also had an analgesic effect along with the joint structure-protective effects. One study showed that both intragastric and intraarticular administration of metformin attenuated articular cartilage degradation and modulated pain-related behavior in DMM-induced OA mouse model [18]. In a monosodium-iodoacetate-induced OA rat model, oral administration of metformin reduced the OA-related pain as well as bone and cartilage damage of the joint [19]. Another animal study reported that oral metformin could inhibit articular cartilage degeneration, synovial hyperplasia, osteophyte formation, and OA-related pain in DMM-induced OA mouse model by activating the AMPK signaling pathway, and the protective effects of metformin on knee OA were also confirmed in non-human primates [20]. The beneficial effect of metformin on OA was also implied by epidemiological studies. A retrospective study reported that OA patients with type 2 diabetes receiving a combination of metformin and cyclooxygenase-2 (COX-2) inhibitors had a lower rate of joint replacement than those receiving COX-2 inhibitors alone [21]. Another prospective cohort study reported metformin usage was associated with a lower rate of medial cartilage volume loss and a trend of reduced risk of total knee replacement in individuals with knee OA and obesity [22].

Thus, experimental and epidemiological evidence suggested that metformin could be repurposed for the management of knee OA. However, there is no clinical trial with high-level evidence supporting this idea. Therefore, we purpose to conduct a randomized, double-blind, and placebo-controlled trial to examine the structure-modifying and symptom-relieving effects of metformin on knee OA.

The objective of this study is to compare, in a randomized, double-blind, and placebo-controlled trial over 24 months, the effect of metformin versus placebo on knee structural changes and knee symptoms in 262 overweight knee OA patients. The primary hypotheses of this trial are that metformin will reduce the loss of tibiofemoral cartilage volume and Western Ontario and McMaster Universities Osteoarthritis Index (WOMAC) score over 24 months compared with placebo in overweight knee OA patients. The secondary hypotheses are that metformin will reduce the visual analogue scale (VAS) knee pain, tibiofemoral cartilage defects, effusion-synovitis volume, and tibiofemoral bone marrow lesions maximum size over 24 months compared with placebo in overweight knee OA patients. If metformin is proved effective, it will offer a novel approach for knee OA treatment.

## Methods/design

### Study design

**Fig. 1:**
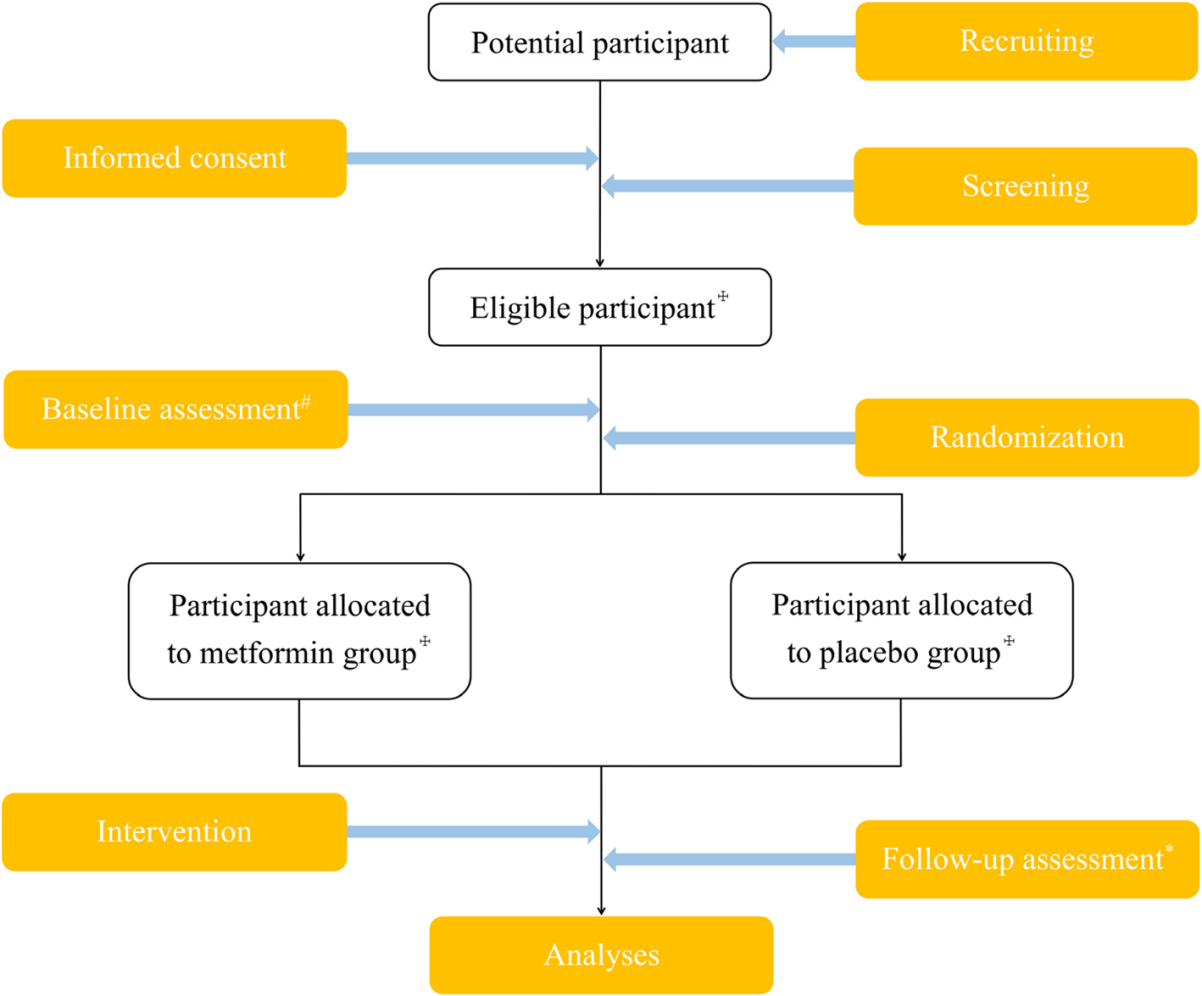
Flowchart of the trial. ^#^MRI, questionnaires, and body composition will be acquired. ^☩^262 participants will be enrolled and equally allocated to the two groups (131 per group) in this trial; however, the number of participants actually enrolled may vary slightly. ^*^The follow-up will be carried out at 3, 6, 12, and 24 months, with questionnaires and body composition being acquired at 3, 6, 12, and 24 months, hematological measurements being acquired at 6, 12, and 24 months, and MRI being acquired at 24 months

This study is designed as a multicenter, randomized, double-blind, placebo-controlled trial over 24 months. Two hundred and sixty-two symptomatic knee OA patients with overweight will be recruited and randomly allocated to either the treatment or placebo control group. The recruitment strategy will include collaborations with specialist rheumatologists, orthopedic surgeons, rehabilitation physicians, and general practitioners, as well as advertising through posters and social media. The primary site of this trial is Guangzhou First People’s Hospital with ethics approval being received from the Ethics Committee of Guangzhou First People’s Hospital (reference number: K-2021-001-02). The sub-sites initially include: Sun Yat-sen Memorial Hospital of the Sun Yat-sen University, Zhujiang Hospital of Southern Medical University, Foshan First People’s Hospital, Liwan Central Hospital of Guangzhou, the Third People’s Hospital of Shenzhen and the Second Affiliated Hospital of Nanchang University. Ethics approval will be received from each of the institutions before the recruitment. Competitive enrollment will be adopted among study sites. Informed written consent will be obtained from all participants. Reporting of the trial will be guided by the Consolidated Standards of Reporting Trials (CONSORT) Statement [23]. The study design of this trial is depicted by Fig. 1.

### Inclusion criteria

1. Meet the American College of Rheumatology (ACR) criteria for symptomatic knee OA [24], assessed by a rheumatologist;
2. Age 50 to 75 years;
3. Body mass index (BMI) ≥ 24 kg/m^2^;
4. Knee pain ≥20 on a 100 mm VAS;
5. Being able to listen, speak, read and understand Chinese, capable of understanding the study requirements and willing to cooperate with the study instructions, and able to provide written informed consent.

### Exclusion criteria

1. Severe radiographic knee OA as grade 3 joint space narrowing according to the Osteoarthritis Research Society International (OARSI) atlas [25];
2. Severe knee pain as knee pain ≥80 on a 100 mm VAS;
3. Planned knee or hip surgery (including arthroscopy, joint replacement, and joint open operation) within 2 years;
4. Severe knee trauma history (including arthroscopy, severe injury of knee ligament or meniscus);
5. Contraindication to magnetic resonance imaging (MRI) scanning (e.g., implanted pacemaker, artificial metal valve or cornea, aneurysm clipping surgery, arterial dissection, metal foreign bodies in the eyeball, claustrophobia);
6. Other forms of inflammatory arthritis (e.g., rheumatoid arthritis, psoriatic arthritis);
7. Active malignant cancer or other life-threatening diseases;
8. Type 1 or type 2 diabetes mellitus;
9. Clinical manifestation of liver dysfunction or elevated alanine aminotransferase/aspartate aminotransferase levels exceeding 2 times the upper limit of normal values;
10. Estimated glomerular filtration rate of less than 60 ml/min/1.73 m^2^;
11. Hypoxic state (e.g., chronic heart insufficiency, acute myocardial infarction, heart failure, chronic obstructive pulmonary disease, cor pulmonale, peripheral vascular disease);
12. Alcoholism;
13. Pregnancy or lactation;
14. Allergic to metformin hydrochloride;
15. Conditions affecting the absorption of oral drugs (e.g., postgastrectomy and malabsorption syndrome);
16. Use of metformin in recent 30 days or plan to use metformin in the next 2 years;
17. Use of investigational drug in recent 30 days.

Note: when both knees of the participants meet the eligibility criteria, the knee with more severe VAS pain will be selected as the study knee.

### Randomization and blinding

To control for variation in study sites, the randomization will be stratified by the study sites. Participants in each site will be randomly assigned to the intervention arm or placebo arm in a ratio of 1:1 based on computer generated random numbers using block randomization (with a block size of 6). Participants, investigators, outcome assessors, as well as statisticians will be blinded to the group allocation in this trial. Allocation concealment will be ensured by keeping the allocation data confidential and using the visual- and taste-masked inert placebo. Emergency unblinding will be permissible when a serious adverse event happens. Participants who are unblinded will be withdrawn from the trial.

### Intervention

Participants in the intervention arm and control arm will take metformin hydrochloride sustained-release tablet (ingredients: microcrystalline cellulose, hydroxypropyl methylcellulose, magnesium stearate, and metformin hydrochloride; Jiangsu Deyuan Pharmaceutical Co., Ltd.) and the identical inert placebo (ingredients: microcrystalline cellulose, hydroxypropyl methylcellulose, magnesium stearate, and lactose; Jiangsu Deyuan Pharmaceutical Co., Ltd.), respectively. The drug and placebo will be administered orally in escalating doses to reduce side-effects and maintain masking: 0.5 g/day for the first 2 weeks, 1 g/day for the next 2 weeks, and then 2 g/day for the remaining period if tolerated. If the subject cannot tolerate the maximum dose (2 g/day), she/he can take their maximum tolerable dose. In case of adverse reaction or deteriorated hepatic and renal function, the researchers can determine whether the subject needs to reduce or stop the drug based on their discretion.

### Quality assurance

Ahead of the recruitment, all research staff will be provided with a standard protocol, a manual of operating procedures (MOP), and a case report form, and will be trained to competently administer all items including questionnaires/surveys, imaging examinations, blood taking, etc. During the study, the project manager will visit each site to monitor trial procedures to ensure the trial is conducted in accordance with the protocol and MOP.

### Outcome measures

Primary outcomes will be changes in tibiofemoral cartilage volume and WOMAC score over 24 months. Secondary outcomes will be changes in VAS knee pain, tibiofemoral cartilage defects, effusion-synovitis volume, and tibiofemoral bone marrow lesions maximum size over 24 months.

#### Assessment of knee structures

Knee structural changes will be assessed using MRI at baseline and 24-month follow-up (Table 1). MRI sequences sensitive for cartilage [e.g., 3D dual-echo in steady state (DESS) or 3D Water Selected fluid (WATSf) sequences] will be used to assess tibiofemoral cartilage volume. MRI sequences sensitive for detailed anatomy [e.g., 2D intermediate-weighted fat suppression or 2D proton density-weighted fat suppression sequences] will be used to assess tibiofemoral cartilage defects, effusion-synovitis volume, and tibiofemoral bone marrow lesions maximum size. All these knee structures will be assessed on the sagittal images. Besides, coronal sequences sensitive for detailed anatomy will be also acquired to assist sagittal images positioning. Details of MRI sequences and parameters will be determined according to the magnetic resonance (MR) scanner available at each site. Before the enrollment, the specific MRI sequences and parameters will be tested in corresponding MR scanners to ensure the MR image meets the study requirement.

**Table 1:** Schedule of data collection

	Screening	months				
		0	3	6	12	24
**Primary outcomes**						
Tibiofemoral cartilage volume		✔				✔
WOMAC		✔	✔	✔	✔	✔
**Secondary outcomes**						
Knee pain VAS	✔	✔	✔	✔	✔	✔
Tibiofemoral cartilage defect		✔				✔
Effusion-synovitis volume		✔				✔
Tibiofemoral bone marrow lesion maximum size		✔				✔
**Other measurements**						
Clinical evaluation and history inquiry	✔					
Joint space narrowing	✔					
Hepatic and renal function	✔			✔	✔	✔
Height	✔					
Weight	✔	✔	✔	✔	✔	✔
Hoffa-synovitis		✔				✔
AQoL-4D		✔	✔	✔	✔	✔
PHQ-9		✔	✔	✔	✔	✔
Body composition		✔	✔	✔	✔	✔
Serum cytokines	✔					✔
Foot pain VAS		✔	✔	✔	✔	✔
Low back pain VAS		✔	✔	✔	✔	✔
Concomitant medication		✔	✔	✔	✔	✔
Pill counts		✔	✔	✔	✔	✔
Adverse events		✔	✔	✔	✔	✔

*WOMAC* Western Ontario and McMaster Universities Osteoarthritis Index, *VAS* visual analogue scale, *AQoL-4D* four-dimensional Assessment of Quality of Life, *PHQ-9* nine-items Patient Health Questionnaire

Cartilage volume will be semi-automatically calculated based on a standardized view of 3D cartilage geometry by OsiriX software (University of Geneva, Geneva, Switzerland). The 3D cartilage geometry is composed from the 2D cartilage shapes, which are generated by drawing contours around the cartilage boundaries on section-by-section MR images [22]. Tibiofemoral cartilage volume will be calculated as the sum of both the tibial and femoral compartments.

Cartilage defects will be graded using a modified Outerbridge classification as follows: grade 0, normal cartilage; grade 1, normal contour but focal blistering and intra-cartilaginous hyperintensity; grade 2, irregularities on the surface with loss of thickness of less than 50%; grade 3, deep ulceration with loss of thickness of more than 50% but no exposure of subchondral bone; grade 4, full-thickness chondral wear with exposure of subchondral bone [26]. Cartilage defects will be assessed at the medial tibial, medial femoral, lateral tibial, and lateral femoral compartments, and tibiofemoral cartilage defects will be obtained by summing the scores of the four compartments.

Effusion-synovitis volume will be measured at suprapatellar pouch, central portion, posterior femoral recess, and subpopliteal recess according to the anatomy of the knee joint synovial cavity. Effusion synovitis will be isolated by selecting a region of interest with an intra-articular fluid-equivalent signal on the section-by-section 2D MR images [26]. The volume of effusion-synovitis will be generated using OsiriX software. Total effusion-synovitis volume of the knee will be obtained by summing the volume of possible effusion synovitis in the four synovial cavities.

Bone marrow lesions is defined as discrete areas of increased signal in the subchondral bone. Bone marrow lesions maximum size will be assessed at the medial tibial, medial femoral, lateral tibial, and lateral femoral compartments. Slice with the greatest area of bone marrow lesions in a specific compartment will be chosen to assess bone marrow lesions maximum size of the corresponding compartment. Bone marrow lesions on adjacent slices will be measured and compared to locate the slice with the maximum lesion size [27]. Tibiofemoral bone marrow lesions maximum size will be calculated by summing the maximum lesions size of the four compartments.

#### Assessment of knee symptoms

Knee symptoms will be assessed by WOMAC score and VAS knee pain at baseline and 3-month, 6-month, 12-month, and 24-month follow-up (Table 1).

The WOMAC system in a 100-mm visual analog format will be used to quantify the degree of knee pain (5 questions), joint stiffness (2 questions), and physical dysfunction (17 questions) during the last 7 days [26]. The WOMAC score will be calculated by summing the score of each question (1 point for every 1 mm), and a higher score of WOMAC represents a more severe OA symptom. The WOMAC score will be considered invalid and treated as missing data if more than 5 of the questions are not answered. In case no more than 5 items are missed, the remaining items will be averaged and then multiplied by 24 to create the WOMAC score.

The 100 mm VAS will be used to assess the knee pain during the last 7 days, and a higher VAS indicates a more severe knee pain.

#### Other measurements

Several other measurements will be performed as follows (Table 1).

Clinical evaluation and history inquiry: clinical evaluation and history inquiry will be conducted for the participant screening.

Joint space narrowing: joint space narrowing will be assessed by a standing anteroposterior semiflexed radiograph of the study knee. X-rays will be scored for joint space narrowing on a four points scale (0-3) using the OARSI atlas [25].

Height: Height will be measured to the nearest 0.1 cm (with shoes removed) using stadiometers.

Weight and body composition: weight and body composition (e.g., fat mass, skeletal muscle mass, and visceral fat mass) will be assessed using bioelectrical impedance analyses.

Hoffa-synovitis: Hoffa-synovitis is defined as discrete areas of increased signal within the infrapatellar fat pad on fat-suppressed water sensitive MRI sequences and will be assessed on the sagittal intermediate-weighted/proton density-weighted sequences according to the following grades: grade 0, none; grade 1, less than 10% of the region; grade 2, 10-20% of the region; and grade 3, more than 20% of the region [28].

Quality of life: The four-dimensional Assessment of Quality of Life (AQoL-4D) questionnaire will be used to assess the quality of life during the last 7 days. AQoL-4D comprises four dimensions of independent living, social relationships, psychological well-being, and physical senses, and each dimension has three items with four levels of severity. The total sum scores range from 0 to 36, and a higher score of AQoL-4D represents a lower quality of life [26].

Depressive symptoms: The nine-items Patient Health Questionnaire (PHQ-9) will be used to assess depressive symptoms during the last 2 weeks. All nine items of the PHQ-9 have four response categories scored from 0 to 3. The total sum scores range from 0 to 27, and a higher score indicates more severe depressive symptoms [26].

Foot pain: The 100 mm VAS will be used to assess the foot pain during the last 7 days, and a higher VAS indicates a more severe foot pain.

Low back pain: The 100 mm VAS will be used to assess the low back pain during the last 7 days, and a higher VAS indicates a more severe low back pain.

Hematological measurements: Fasting morning blood samples will be collected at the screening and 6, 12, 24-month follow-up. Serum levels of alanine aminotransferase and aspartate aminotransferase and estimated glomerular filtration rate will be tested at the above time points to reflect the hepatic and renal function. Besides, at the screening and 24-month follow-up, blood lipid profile and C-reactive protein will be tested and serum will be isolated and stored at − 80 °C for further measurement of inflammatory cytokines (e.g., tumor necrosis factor**-**α, interleukin-1, interleukin-6) and adipokines (e.g., leptin, resistin, visfatin).

Concomitant medication: Concomitant medication usage will be not restricted in this study, but will be documented at the baseline and each follow-up.

Pill counts: The pill counts will be recorded to infer the medication compliance of the participants.

### Safety assessment

Adverse events of metformin will be closely monitored at each visit during the study (Table 1). The participants will be requested to report any adverse events to the research staff spontaneously. Details of the adverse event and its relationship with the study intervention will be recorded.

### Sample size

The sample size calculations were performed using the formula: n_1_ = n_2_ = 2 × [(Z_α_ + Z_β_) × σ/δ]^2^ and assumed α = 0.025 (one-sided) and β = 0.20 (Z_α_ = 1.96; Z_β_ = 0.842).

According to a previous study, the standard deviation of annual tibiofemoral cartilage volume loss in obese knee OA patients was 1.92% (σ), and taking metformin could decrease the average annual tibiofemoral cartilage volume loss by 1.07% (δ) [22]. Considering that it may take a long time for metformin to exert the protective effect on articular cartilage, we assumed that the volume of articular cartilage begins to differ after 1 year of metformin use, that is, the second year of follow-up can be used to observe the effect of metformin on the articular cartilage volume. Based on the assumption and the data from the previous study, we calculated that 51 participants per group (n_1_, n_2_) are needed.

Sample size calculation was also performed based on another primary outcome, namely, WOMAC score. Up to now, no study has reported the effect of metformin on WOMAC score. We hypothesized that metformin treatment for 2 years will have more decrease in WOMAC score compared with placebo. The minimum clinically important difference (MCID) of WOMAC score in patients with knee OA was reported being 12, 14, 16, 18, and 20% of the baseline score [29–33]. Combined, we took the 16% for sample size calculation. In our previously established Chinese symptomatic knee OA cohort, the mean baseline WOMAC score was 99.95 (a total score of 240) [28]. Based on these data, it is estimated that the difference in the changes in WOMAC score (a total score of 2400) between metformin and placebo groups needs to be greater than 159.92 (δ) to achieve MCID. In our previous clinical trials for patients with knee OA, the standard deviation of change from baseline to two-years follow-up of WOMAC score was 413.74 (σ). Therefore, the sample size was calculated as 105 for each group (n_1_, n_2_).

Take into consideration of these two calculations and a 20% dropout over the trial, 131 participants in each arm will be sufficient to detect the differences of primary outcomes between the two groups.

### Statistical analysis plan

Baseline characteristics will be reported per group using descriptive statistics (means, proportions, and medians as appropriate) without statistical tests.

The primary analyses will be intention-to-treat analyses of primary and secondary outcomes. Per-protocol analyses will be performed as the secondary analyses, and participants with an average daily dose of 0.5-2 g of the investigational drug will be included in the per-protocol analyses. Missing data caused by loss to follow-up and nonresponses will be addressed by multiple imputations with chained equations. Imputations will be performed separately for each treatment group and each outcome using baseline variables of age, sex, BMI, and study site and non-missing values of the outcomes at baseline and each follow-up with the assumption of data missing at random. The treatment effect will be analyzed using linear mixed effect models. In the models, baseline covariates (age, sex, BMI, baseline value of the corresponding outcome), treatment, month, and the interactions of covariates and treatment with month will be entered as fixed effects. The correlation within the study sites and the repeated measures will be addressed using trial site and participant identification as random intercepts. Month will be treated as random slope to allow different treatment effects among knee OA patients over time. Changes in outcome measures within each group and difference of the changes between groups from baseline to follow-up will be calculated using linear combinations of the estimated coefficients.

Pre-specified stratified analyses will be performed to explore whether BMI could affect the treatment effect of metformin on knee OA. This will be conducted by introducing a 3-way interaction between treatment, month, and BMI in the linear mixed-effects models.

Because of the potential for type I error due to multiple comparisons, the findings for the analyses of the secondary outcomes and the stratified analyses will be interpreted as exploratory. A 2-sided *P* value of 0.05 or less will be considered as statistically significant.

### Withdrawal

If participants withdraw from the study before the end of the trial, the reason and date will be recorded. Participants who withdraw from the trial after 6 months of follow-up will be asked to have the final assessments at the 24-month follow-up. Outcomes assessed before the withdraw and at the 24-month follow-up will be used for the statistical analyses.

### Data integrity and management

All data obtained will be kept strictly confidential and will be stored electronically on a database with secured and restricted access (Research Electronic Data Capture, REDCap). After each follow-up, the obtained data will be timely inputted into the electronic database. The entered data will be routinely checked to correct the potential mistaken input. The obtained MR images will be stored electronically and transferred to the sponsor (Guangzhou First People’s Hospital) in time. At the completion of the follow-up, the MR images will be uniformly assessed and the acquired data will be inputted into the electronic database at the primary site. Then, all the data will be checked and locked. After that, a two-step unblinding method will be applied. The first step of the unblinding will classify the participants to group A and group B for efficacy endpoints and safety analyses. When the analyses are completed, the second step of the unblinding will be conducted to assign group A and group B.

## Discussion

We have proposed a multicenter, randomized, double blind, and placebo-controlled trial to determine whether metformin intervention can modify knee structures and relieve knee symptoms in overweight knee OA patients. If metformin is proved to be effective on knee structures and symptoms, it will be a novel therapeutic approach to reduce knee OA progression.

Metformin is not just an antihyperglycemic drug but also has multiple health-protective effects [34, 35]. In vivo studies found that metformin had both structure-modifying and symptom-relieving effects in knee OA animal models [18–20]. Epidemiological studies also suggested a protective effect of metformin on OA [21, 22]. For the underlying mechanisms, studies suggested that the beneficial effect of metformin on OA could be mediated by protecting chondrocytes and inhibiting pain [18–20]. Also, it is reported that metformin could inhibit the inflammatory response in macrophages, while hyperinflammatory synovial macrophages exacerbate the progression of OA [36, 37]; therefore, metformin may exert OA-protective effect by modulating the inflammatory response of macrophages.

Obesity is an important risk factor for knee OA, as obesity increases not only the loading but also the pro-inflammatory adipokines, leading to mechanical damage as well as metabolic imbalances of the joint [38, 39]. Metformin has been shown to have a weight-losing effect [12], which could also explain the potential OA-protective effect. Our study found that metformin had a stronger protective effect against OA in obese OA mice than in OA mice with normal weight (not yet published). Therefore, overweight knee OA patients were selected in this trial as these knee OA patients are more likely to benefit from metformin intervention than those with normal weight. Besides, pre-specified analyses of interactions between BMI and metformin intervention on OA outcomes will be performed to explore if knee OA patients with higher BMI could benefit more from metformin intervention.

To assess OA progression, accurate measurement tools which could evaluate cartilage morphology and other structures of the joint are needed. Radiographic OA assessment is not responsive to a tangible change over a short interval, and as a two-dimensional assessment, it is highly vulnerable to measurement error because of issues such as variations in joint positioning [40]. MRI allows direct, accurate, and reliable assessment of joint structures, therefore offers a much better assessment for detecting knee OA progression [40, 41]. MRI assessment of cartilage morphology is now recommended as an endpoint for the evaluation of structure modification in trials of knee OA [41]. Therefore, cartilage volume as a continuous variable which can predict total knee replacement was selected as a co-primary outcome in this trial. Simultaneously, the WOMAC score which is the most commonly-used disease-specific outcome in OA [26] was selected as another co-primary outcome for the evaluating of OA symptoms. Thus, the findings from this study will show whether metformin intervention has both structure-modifying and symptom-relieving effects on knee OA. As for the secondary outcomes, structural changes including cartilage defects, effusion-synovitis, and bone marrow lesions are all predictive of total knee replacement [42–44], suggesting they are clinically relevant. VAS knee pain was also selected as a secondary outcome since it is thought to be the gold standard method for quantifying arthritic pain [45].

At present, knee OA is a major public health problem without effective therapeutics. Animal experiments and observational studies have suggested that metformin may have protective effects on knee OA. Based on these previous findings, this trial has been designed to determine whether metformin intervention can slow the progression and relieve the symptom of knee OA. If metformin is proved to be effective for knee OA, it will be used as a disease-modifying OA drug and therefore reduce the economic burden of the disease through improving the quality of life as well as reducing the need of joint replacement for knee OA patients.

## Data Availability

Not applicable.

## Abbreviations

ACR: American College of Rheumatology
AMPK: AMP-activated protein kinase
AQoL-4D: Four-dimensional Assessment of Quality of Life
BMI: Body mass index
CONSORT: Consolidated Standards of Reporting Trials
COX-2: Cyclooxygenase-2
DESS: Dual-echo in steady state
DMM: Destabilization of the medial meniscus
GDP: Gross domestic product
mTOR: Mammalian target of rapamycin
MR: Magnetic resonance
MRI: Magnetic resonance imaging
OA: Osteoarthritis
OARSI: Osteoarthritis Research Society International
PHQ-9: Nine-items Patient Health Questionnaire
SIRT1: Specifically silent information regulator 1
VAS: Visual analogue scale
WATSf: Water Selected fluid
WOMAC: Western Ontario and McMaster Universities Osteoarthritis Index
